# The influence of mixing international and domestic students on competency learning in small groups in undergraduate medical education

**DOI:** 10.1186/s12909-020-02277-0

**Published:** 2020-10-08

**Authors:** Yan Zhou, Agnes D. Diemers, Jasperina Brouwer, Friso L. H. Muntinghe, Robbert J. Duvivier, Jan Pols, A. Debbie C. Jaarsma, Nicolaas A. Bos

**Affiliations:** 1 Institute for Medical Education, University of Groningen, University Medical Center Groningen, Groningen, The Netherlands; 2 Educational Sciences, Faculty Behavioural and Social Sciences, University of Groningen, Groningen, The Netherlands; 3 Department of Internal Medicine, University of Groningen, University Medical Center Groningen, Groningen, The Netherlands; 4 Parnassia Psychiatric Institute, The Hague, The Netherlands

**Keywords:** Learning community, Competency-based medical education, Internationalization

## Abstract

**Background:**

Medical curricula are increasingly internationalized, with international students being mixed with domestic students in small group learning. Small group learning is known to foster competency learning in undergraduate medical education, specifically Communication, Collaboration, Leadership, and Professionalism. However, it is unclear what happens with the learning of competencies when international students are introduced in small groups. This study explores if students in international small groups master the competencies Collaboration, Leadership and Professionalism at the same level as students in domestic groups in an undergraduate medical curriculum.

**Method:**

In total, 1215 Students of three academic year cohorts participated in the study. They were divided into four learning communities (LCs), per year cohort, in which tutor groups were the main instructional format. The tutorials of two learning communities were taught in English, with a mix of international and Dutch students. The tutorials of the other two learning communities were taught in Dutch with almost all domestic students. Trained tutors assessed three competencies (Collaboration, Leadership, Professionalism) twice per semester, as ‘Not-on-track’, ‘On-track’, or ‘Fast-on-track’. By using Chi-square tests, we compared students’ competencies performance twice per semester between the four LCs in the first two undergraduate years.

**Results:**

The passing rate (‘On-track’ plus ‘Fast-on-track’) for the minimum level of competencies did not differ between the mixed and domestic groups. However, students in the mixed groups received more excellent performance evaluations (‘Fast-on-track’) than the students in the homogenous groups of Dutch students. This higher performance was true for both international and Dutch students of the mixed groups. Prior knowledge, age, gender, and nationality did not explain this phenomenon. The effect could also not be explained by a bias of the tutors.

**Conclusion:**

When students are educated in mixed groups of international and Dutch students, they can obtain the same basic competency levels, no matter what mix of students is made. However, students in the mixed international groups outperformed the students in the homogenous Dutch groups in achieving excellent performance scores. Future research should explore if these findings can be explained from differences in motivation, perceived grading or social network interactions.

## Background

Health professions education in the twenty-first century is facing complex challenges [1]. A Lancet commission, consisting of 20 professional and academic leaders from different countries constructed a vision to drive major reforms in health professions education as an answer to global problems, such as health inequity between countries, demographic and epidemiological changes, growth of knowledge and technology, and complex care, either home-based or community-based [2]. They propose a framework in which both the Education system and Health system are driven by the needs of the population, which in turn elicit demands for educational and health services. Concerning the educational system, they identify three key dimensions: institutional design, instructional design and educational outcomes, which are all three influenced by local as well as global contexts [2]. In order to be able to catch up with the aforementioned global developments, it is important that curricula focus not only on local health care problems but also take global health care into consideration [3]. Moreover, students increasingly show interest in learning and working in different context situations all over the world, and make use of international exchange programmes [4]. Therefore, globalization and internationalization of health care and health professionals should be integrated in education.

As for the key dimension ‘instructional design’, the commission advocates competency based curricula that offer students different learning activities and methods to master competencies, for use in local as well as global contexts, and that prepare them for life-long learning [2]. During the last decades, competency-based medical education (CBME) has increasingly been introduced in graduate and undergraduate medical education [5, 6]. CBME can be based on different competency frameworks that are derived from analysis of societal and patient needs and prepares future physicians for their professional work [7, 8]. The purpose of using CBME is to ensure that physicians are equipped with a sufficient level of basic competencies [9]. It is known that small group learning fosters the learning of competencies [10, 11]. Different instructional designs use small group learning in undergraduate medical education. One example is Problem Based Learning (PBL) [12–14]. PBL is known to facilitate students’ Communication, Collaboration, and Leadership competencies development [15–17]. Another example of an instructional design that uses small group learning is the use of Learning Communities (LCs), which are known to improve interaction and relationships between students and faculty [18]. Students construct knowledge by interacting with fellow students and create meaningful connections between their experience and classwork [19]. In a LC, as in PBL, students meet regularly to collaborate on classwork which benefits both their experience, sharing and professional competency development [9]. So, PBL and LC both seem to foster competency learning in undergraduate medical education, specifically Communication, Collaboration, Leadership, and Professionalism.

Parallel to the globalization of Health Professions Education, there is a movement to increasing internationalization in higher education in general [3, 4]. This can take several forms, but the main tenet is to integrate an intercultural or global dimension into student learning. Many universities have actively attracted students from other countries to diversify cohorts, so that domestic students may gain intercultural experience without leaving the country [20]. Some medical schools use professional development spine (PDS) tutor group, small group, peer community, virtual collaboration or campus influence to improve international interactions among students by allocating them randomly into groups [21–25]. This is a relatively new phenomenon in medicine, which has been researched mainly from the point of view of the international students. Studies have shown many universities still face the challenges that cover the cross-cultural environment and curricular adaptations [3].

In order to advance globalization, medical curricula also want to admit international students to their competency-based curricula, using small group learning. Although the benefits of small groups on competency learning are known, we do not know what happens with the learning of competencies when international students are introduced in small group learning. Do they acquire the same competencies and on the same level as students in domestic small groups? Therefore, with this study we want to explore if students in mixed international small learning groups master the competencies of Collaboration, Leadership and Professionalism at the same level as students in domestic small learning groups in a PBL- and LC-based undergraduate medical curriculum over time.

## Methods

### Educational background

**Fig. 1:**
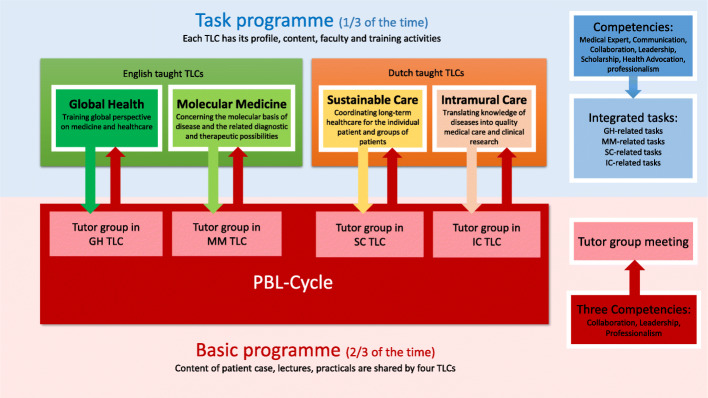
The curriculum design of G2020

In the Netherlands, undergraduate medical education takes six years, divided into a bachelor phase (the first three years) and a master phase (the next three years) [26]. Since 2014, the University Medical Center of Groningen (UMCG) has a bachelor curriculum named G2020, designed according to PBL activities within four learning communities (LCs) [27]. G2020 adopts the CanMEDS framework, which is the most commonly used and integrated model in medical education worldwide. This framework focusses on seven competencies: Medical Expertise, Communication, Collaboration, Leadership, Health Advocacy, Scholarship and Professionalism [28–34]. The bachelor curriculum G2020 includes a basic programme and a task programme, covering about two-third and one-third of the time, respectively. The basic programme is identical for all four LCs while the task programme is specific for every LC and consists of integrated tasks (Fig. 1). In the basic program, learning material and faculty are the same for all LCs; only the language of instruction differs according to the type of LC. However, in the task program, each LC has its own profile, aims, content, faculty and training activities. The profiles of these four LCs reflect future healthcare developments: Sustainable Care (SC), Intramural Care (IC), Global Health (GH), and Molecular Medicine (MM). LC Sustainable Care and Intramural Care are Dutch taught, and students are almost all Dutch. LC Global Health and Molecular Medicine are English taught, since a mix of Dutch and international students are involved. During the selection procedure before the start of the study, students choose their LCs based on their own academic interests and language preferences. All students join in one LC for the entire bachelor phase.

One academic year has two semesters and each semester has two blocks of ten weeks each. The basic programme consists of PBL cycles (see Fig. 1) and tutor groups. Every week the cycle starts with a lecture dealing with a live patient interview. Consecutively, students meet in tutor groups of ten students from the same LC twice a week. In the first meeting, the students work on the patient problem that was presented in the lecture at the beginning of the week, to acquire and activate prior knowledge [13]. Students discuss how to deal with that case and discover gaps in their knowledge. After the meeting, students need to fill their knowledge gaps by studying literature and supporting learning activities, such as lectures, seminars, and practical exercises. In the next tutor group meeting, students share what they have learned. Thus, the tutor group is a pivotal part of the basic programme and organized for students from the same LC. In the tutor group basic knowledge and specific LC profile knowledge are integrated. The assessment takes place at the end of each block. In the tutor group, students only train three competencies that closely relate to tutor group performance: Collaboration, Leadership, and Professionalism. Collaboration aims to evaluate how students contribute to the group meeting and if they work effectively with other students, share information, and reply to each other’s questions. Leadership focuses on students’ performances when they act as a group leader, how they guide and take responsibility for the group, and contribute to good outcomes of the group. Professionalism examines students’ standards of behaviour and their specialist knowledge usage [28]. These three competencies, as reflected by their performance in meeting organization, discussion, and personal presentations, are assessed by the tutors (so-called tutor group assessment). Tutors are trained Master students, who are assigned randomly to the tutor groups and change every block.

Different from the basic programme, the task programmes are variable, reflecting the different designs of the LCs. The task programme aims for the development of all seven CanMEDS competencies. Different forms of activities are used to perform or support the tasks, like role play, mock consultation, expert meeting, emulation scene interaction, project demonstration, and personal presentations. Each task assesses two to three competencies and all seven competencies are assessed at least twice in every LC per year. Students in the task programme are assessed by long-term coaches, occasional experts, peers, or through self-evaluation.

### Participants

Medical bachelor (BA) students participated of the year cohorts 2014–2015 (BA1415), 2015–2016 (BA1516) and 2016–2017 (BA1617) of the University Medical Center of Groningen (UMCG) in the Netherlands. The numbers of each cohort varied between 393 and 420 participants across the years (*N* = 1215, 68% female, 32% male, see Table 1). The mean age of all participants was 19.14 years (*SD* = 1.652). The majority of students were Dutch (*N* = 1024; 84%), of which 663 (65%) participated in one of the two Dutch taught LCs and 361 (35%) participated in one of the two English taught LCs. Among the 191 international students (16%), 5 students (3%) participated in a Dutch LC, whereas 186 (97%) participated in an English LC.

**Table 1:** Description of participants of all cohorts

Cohorts	Language	LC	Student Number	Gender		Age	Nationality	
				Male	Female	(mean)	Dutch	International
BA1415	Dutch	SC	75	26	49	19.35	75	0
		IC	121	36	85	18.88	120	1
	English	GH	105	36	69	19.54	79	26
		MM	92	36	56	19.21	69	23
		**Total**	**393**	**134**	**259**	**19.22**	**343**	**50**
BA1516	Dutch	SC	98	20	78	18.72	96	2
		IC	144	42	102	18.94	144	0
	English	GH	86	23	63	19.64	45	41
		MM	74	26	48	19.30	50	24
		**Total**	**402**	**111**	**291**	**19.10**	**335**	**67**
BA1617	Dutch	SC	73	22	51	19.01	72	1
		IC	157	48	109	18.76	156	1
	English	GH	89	29	60	19.25	53	36
		MM	101	42	59	19.56	65	36
		**Total**	**420**	**141**	**279**	**19.10**	**346**	**74**
Total			1215	386	829	19.14	1024	191

### Measurements

To minimize the bias caused by differences in the programmes of the LCs regarding students’ competencies performance assessment, we analyzed tutor group assessments on Collaboration, Leadership and Professionalism of the basic programme. The curriculum design, learning materials and the type of assessor of this part of the basic programme are the same for all LCs. The design of the task programme varies substantially, so the results of competency assessments in those LC programmes cannot be compared easily. We decided to analyze only those three competencies, because these were the ones assessed in the shared basic program.

The basic programme uses a three-scale score to measure competency performance: Fast-on-track, On-track and Not-on-track. Fast-on-track means students pass the assessment and perform excellent in the evaluation, On-track means students pass the assessment and perform at the average level, and Not-on-track means that students fail in that evaluation. The reason for the three-scale scoring is based on the assumption that competencies are best assessed by many low stake evaluations resulting in a cumulative high-stake decision [35]. At the end of each block (four times a year), students’ Collaboration, Leadership and Professionalism competencies were assessed by the tutors with the explanation of their score results. Thus, across the first two years, students were graded eight times. This means that for each competency the total number of each score (Fast-on-track, On-track and Not-on-track) varies from 0 to 8. The total number of each score across two years is calculated as a count variable based on the three competencies across 8 time points (range from 0 to 24).

In order to better understand the impact of the LC on students’ results on the three key competencies, some additional data were collected regarding possible factors influencing the outcomes: students’ age, gender, nationality and their scores on the first progress test. The progress test is a curriculum-independent test [36] and is used to assess students’ medical knowledge four times a year during their whole curriculum. The first progress test is taken directly after the start of the first semester of Bachelor year 1 and is assumed as students’ prior medical knowledge assessment in the current study. The progress test consists of 200 multiple choice questions with two-, three-, or four answering categories. Depending on the answering categories, they obtain points for a good answer. When they select the additional answering category ‘I don’t know’, they do not obtain points and when the answer is wrong, points will be subtracted.

### Data collection

Students’ competency assessment results and their background information were collected from several databases from the administration office of the UMCG Medical Faculty. One independent database manager integrated all data, encrypted the data and provided the anonymous data to the researchers. This study collected the first and second-year results of tutor group assessments for all cohorts. The study was approved by the Ethical Review Board of the Netherlands Association of Medical Education (NVMO), dossier number 2019.4.8.

### Statistical analyses

The analysis is performed in different steps. First, we calculated student’s total numbers and percentages of Not-on-track, On-track, and Fast-on-track of the competencies Collaboration, Leadership, and Professionalism obtained across two bachelor years. This exploration provided a first insight in the frequency of the scores and the differences descriptively among LCs and the three competencies.

Second, since the students’ competencies assessment results (the total number of Not-on-track, On-track, and Fast-on-track every students obtained across two years) were not normally distributed, the nonparametric Kruskal-Wallis *H* test was performed to test whether respectively the Not-on-track, On-track and Fast-on-track score on the three competencies of Collaboration, Leadership, and Professionalism differs between the four LCs. The average number of scores each student obtained in two years was compared among LCs.

Third, Chi-square tests were performed on students’ competencies score separately over time (8 blocks) to address the research question of whether students in mixed international small learning groups achieve the same level of competencies Collaboration, Leadership and Professionalism compared to students in domestic small learning groups in different LCs during the first two years of the bachelor programme over time.

Fourth, we explored the difference of competencies performance between international students and domestic students in the mixed international LCs by comparing the percentage of times students obtained Fast-on-track in all assessment across two years.

Fifth, the relationship between the total number of times students got Fast-on-track on the three competencies Collaboration, Leadership, and Professionalism across two years, the first progress test, and background characteristics were assessed by Spearman rank correlation analyses. Besides, the Kruskal-Wallis *H* test was performed to explore if students’ prior knowledge (first progress test) differs across LCs. The scores of the first progress tests were not normally distributed either, although the dependent variable (first progress test) has a continuous scale. The SPSS software 25.0 programme was used for all statistical analyses.

## Results

### Descriptive statistics

Students of the four LCs were evaluated by their tutors for the three competencies Collaboration, Leadership and Professionalism at the three-scale score Not-on-track, On-track and Fast-on-track (Table 2). The percentage of students in all four LCs who passed assessment (Fast-on-track plus On-track) on all three competencies is higher than 98%. The percentage of students who obtained Not-on-track on the three competencies ranged from 0.54 to 1.99%. The LCs did not significantly differ for the number of Not-on-track students across two years (*H* (3) = .907, *p* = .824). In contrast, the percentage of students who obtained On-track and Fast-on-track between international and domestic LCs were different. The majority of all students’ competencies assessment results (over 57%) was on track (On-track), but the percentage of students in the mixed international LCs (Global Health and Molecular Medicine) who obtained Fast-on-track were higher than in the domestic LCs (Sustainable Care and Intramural Care), around 40 and 30% respectively. The differences of these percentages within the same language LCs (respectively between Global Health and Molecular Medicine and between Sustainable Care and Intramural Care), range from 0.48 to 1.35%. When comparing the percentage of Fast-on-track between the three competencies in the four LCs, Leadership almost always had the highest percentage and Professionalism had the lowest percentage of Fast-on-track, which means students are more easily able to get Fast-on-track on Leadership than on Professionalism.

**Table 2:** Students’ competency assessment results across LCs

LC	Competency	Not-on-track	On-track	Fast-on-track
		N/Total N (%)	N/Total N (%)	N/Total N (%)
SC	Collaboration	15/1672 (0.90%)	1142/1672 (68.30%)	515/1672 (30.80%)
	Leadership	15/1672 (0.90%)	1079/1672 (64.53%)	578/1672 (34.57%)
	Professionalism	29/1672 (1.73%)	1204/1672 (72.01%)	439/1672 (26.26%)
	**Total**	**59/5016 (1.18%)**	**3425/5016 (68.28%)**	**1532/5016 (30.54%)**
IC	Collaboration	31/2676 (1.16%)	1827/2676 (68.27%)	818/2676 (30.57%)
	Leadership	23/2676 (0.86%)	1762/2676 (65.84%)	891/2676 (33.30%)
	Professionalism	41/2676 (1.53%)	1931/2676 (72.16%)	704/2676 (26.31%)
	**Total**	**95/8028 (1.18%)**	**5520/8028 (68.76%)**	**2413/8028 (30.06%)**
GH	Collaboration	10/1860 (0.54%)	1080/1860 (58.06%)	770/1860 (41.40%)
	Leadership	15/1860 (0.81%)	1045/1860 (56.18%)	800/1860 (43.01%)
	Professionalism	37/1860 (1.99%)	1102/1860 (59.25%)	721/1860 (38.76%)
	**Total**	**62/5580 (1.11%)**	**3227/5580 (57.83%)**	**2291/5580 (41.06%)**
MM	Collaboration	25/1724 (1.45%)	1051/1724 (60.96%)	648/1724 (37.59%)
	Leadership	17/1724 (0.99%)	954/1724 (55.34%)	753/1724 (43.68%)
	Professionalism	18/1724 (1.04%)	1053/1724 (61.08%)	653/1724 (37.88%)
	**Total**	**60/5172 (1.16%)**	**3058/5172 (59.13%)**	**2054/5172 (39.71%)**

### Competency assessment between four LCs over time

**Fig. 2:**
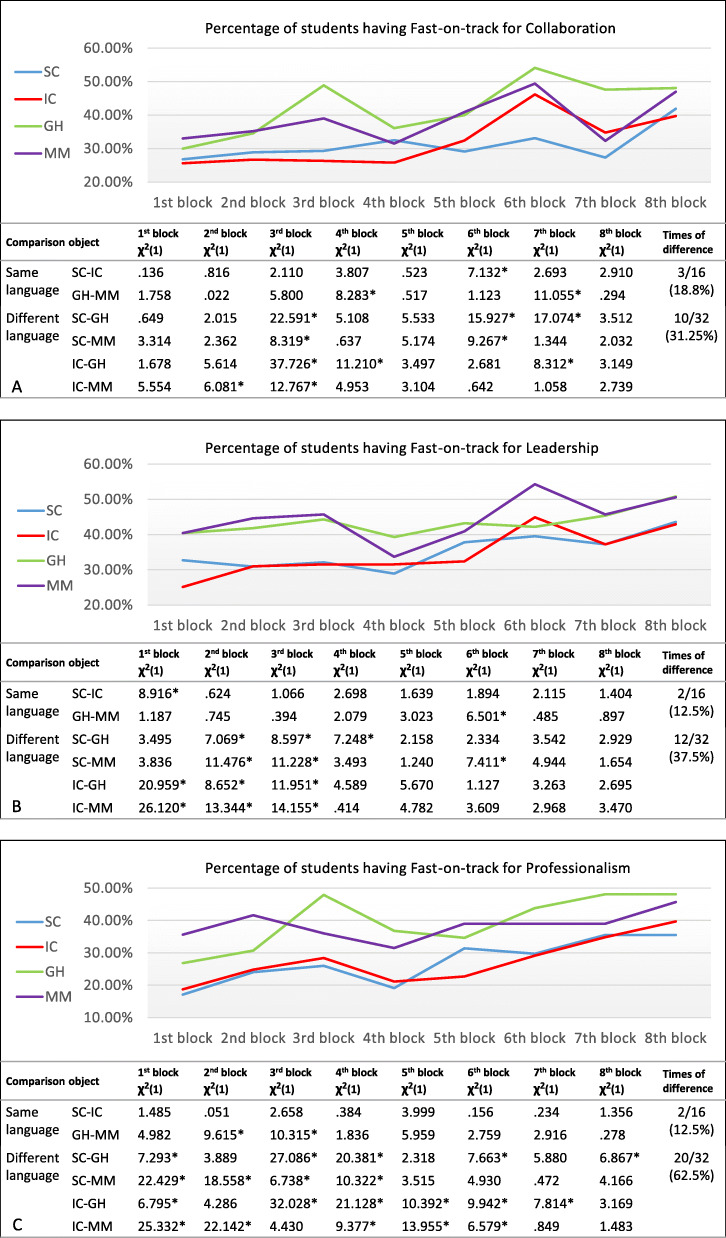
Students’ competencies Collaboration (**a**), Leadership (**b**), and Professionalism (**c**) performance of four learning communities over time. The line chart presents the percentage of students with a Fast-on-track within four learning communities, with blue line = Sustainable Care (SC), red = Intramural Care (IC), green = Global Health (GH) and purple = Molecular Medicine (MM). Below the figure Chi-square test results of the comparisons of students scores (Fast-on-track, On-track and Not-on-track) are shown between every two LCs in every block, and the number of times (and percentages) that significant differences were found in the same language LCs and different language LCs are shown in the last column. Same language means the study language in the two LCs are the same, all in English or all in Dutch

Even though the passing rates of the three competencies assessments for students were similar among the four LCs (around 98%), the LCs did significantly differ regarding the number of students obtaining On-track (*H* (3) = 64.98, *p* < .001) and Fast-on-track (*H* (3) = 117.321, *p* < .001). The average numbers of each student obtaining Not-on-track in four LCs are low, range from 0.23 to 0.25. The average numbers of students obtaining Fast-on-track in two domestic LCs are 7.43 (*SD* = 4.537) and 7.43 (*SD* = 4.160) respectively and in two international LCs are 10.12 (*SD* = 4.754) and 9.79 (*SD* = 4.108) respectively. The average numbers of students obtaining On-track in two domestic LCs are 16.32 (*SD* = 4.382) and 16.33 (*SD* = 4.039) respectively and in two international LCs are 13.65 (*SD* = 4.596) and 13.99 (*SD* = 3.921) respectively. Students’ competencies scores were further analyzed regarding the extent to which students from international and domestic LCs differ in their three key competencies performance over time. Students’ competencies performance between the four LCs in the first two bachelor years were evaluated (Fig. 2a, b, c).

In all significant differences found between domestic and international LCs in two years (*N* = 42), international LCs always (*N* = 41) have significantly more students obtained Fast-on-track and significantly fewer students obtained On-track than domestic LCs, with no significant difference on Not-on-track. Only once LC Molecular Medicine has significantly more students obtained Not-on-track than LC Intramural Care, but LC Molecular Medicine still has significantly more students obtained Fast-on-track and significantly fewer students obtained On-track than LC Intramural Care. Focusing on the higher-achieving students, the percentage of Fast-on-track that students in each LC received illustrate an increasing trend over eight blocks (two years). After two years, the percentage of students that received a Fast-on-track was higher in all LCs, although this increase varied over time. The total percentage of students with a Fast-on-track increased from 28 to 43% for Collaboration, from 33 to 46% for Leadership and from 25 to 42% for Professionalism during the two academic years. Meanwhile, different LCs and competencies showed different trajectories. For Collaboration assessment among the four LCs, LC Global Health fluctuates the most while LC Sustainable Care is most stable but steadily increasing. The two international LCs always showed a higher percentage of Fast-on-track than the two domestic LCs in Professionalism assessment, while they sometimes showed lower percentages of Fast-on-track than the two domestic LCs in Collaboration and Leadership assessment.

**Fig. 3:**
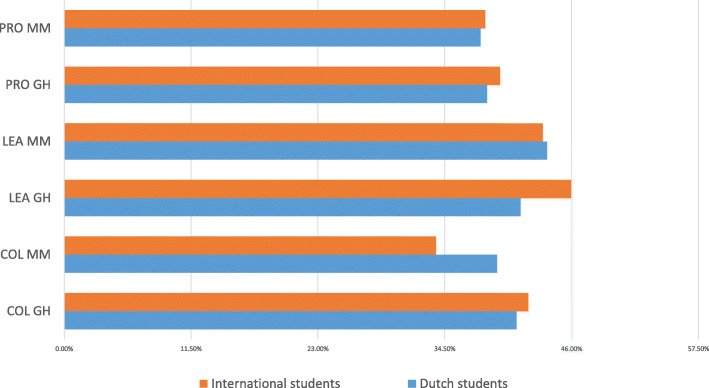
The percentages of excellent performance (Fast-on-track) of Dutch and International students in English LCs

To find out whether the international students alone caused the observed differences between the Dutch and the English LCs [37], a sub-analysis was performed for the Dutch and International students within LC Molecular Medicine and LC Global Health separately. The number of times Fast-on-track was obtained by domestic and international students were similar (see Fig. 3). Nationality did not cause the Fast-on-track difference between the English LCs and Dutch LCs.

### Post-hoc spearman rank correlation analysis

To consider what factors could have influenced the observed higher percentage of Fast-on-track between the mixed international LCs and domestic LCs, associations were explored with Spearman rank correlation analysis between students’ competency performances (Fast-on-track), background (age, gender, and nationality), and prior medical knowledge (see Table 3 in Appendix). The Fast-on-track performances of the three competencies moderately correlated with each other. Students’ backgrounds and prior medical knowledge showed weak or very weak correlation with their competency performance. A comparison of students’ first progress test between four LCs showed no significant difference *(H* (3) = 3.80, *p* = .284*)*. This implies that the students did not differ in their medical knowledge at the start of their medical programme.

## Discussion

This explorative study provides empirical information that it is possible to train students to reach the same basic level (to pass assessment) of competencies when international students are mixed with domestic students in LCs. We observed that all students had higher percentages of excellent performance (Fast-on-track) at the end of the second year compared with the first year. However, students in mixed international LCs received significantly more Fast-on-track evaluations than the students in the domestic LCs. This was true both for the domestic students as well as for the international students within the mixed international LCs and was independent of the students’ nationalities. Domestic students and international students performed similarly in mixed international LCs and both received significantly more excellent performance evaluations than students in the domestic LCs. Age, gender, and prior knowledge had no association with the observed differences either, although it is known that these factors may correlate with students’ performance [38]. Also, possible bias of the assessors is not likely, since tutors were randomly distributed among tutor groups in different LC’s every block.

How can we explain why domestic students with similar prior knowledge performed differently in mixed international LCs and domestic LCs while domestic students and international students performed similarly better in mixed international LCs? It has been shown that students tend to assimilate the average academic performance of their peers, the so-called ‘peer effect’ [21, 39]. According to the social comparison theory, people have the drive to evaluate their own performance, motivation and ability by comparing themselves to others and then change their behavior accordingly [40–42]. Thus, learning communities may create such a special environment for students. Students in the same LC could be influenced by ‘peer effect’ and possibly kept comparing their own performances with peers during the study process. It may explain why students in the same LC performed similarly but differ from students in other LCs, although they all have similar prior knowledge.

Although students might compare their results to peers within the same LCs, this still does not explain the differences between the international and domestic LCs. One possible explanation could be a difference in student’s motivation. It has been described that student’s motivation influences academic achievement [43]. Students’ competencies performance in the mixed international LCs could possibly be influenced by both intrinsic and controlled motivation—a type of motivation that comes from external rewards or fear of punishment [44]. For instance, the grading systems in the Netherlands and other countries are different. In Anglo-Saxion systems, score B and C (comparable with On-track), especially C, may be considered as bad scores whereas On-track is a common score in the Dutch system. For international students, they may be used to get Fast-on-track rather than On-track, so their intrinsic motivations influence their study process in this case. Some international students may put pressure on themselves to perform better to get Fast-on-track. Moreover, international students may even cause social pressure to their group members and tutors within the small group environment with regard to performance [42]. Thus, the performance of both international and domestic students in mixed international LCs may develop similarly resulting in more students having Fast-on-track qualifications. Another factor that could play a role is that domestic students who study in mixed international LCs use their second language to study, which is more difficult than using their native language. Consequently, domestic students who choose mixed international LCs may have higher intrinsic motivation than other domestic students, reflecting in better performance of competencies.

Besides the above described ‘peer effect’ and the role of motivation, students’ interpersonal relationships, such as help-seeking relationships, fellow students’ support, and friendship, also may influence students’ academic performance [21, 45, 46]. It is known that the dynamic and degree of closeness of students’ interpersonal relationships predicts similarity in academic achievement to some extent [21, 47, 48]. It is possible that students in mixed international LCs feel more relatedness with their peers in the same LC and are more eager to make a connection with each other for not only study-related support but also for friendship networks. As a result, students in mixed international LCs may have more dynamic and extensive social networks than in domestic LCs. Even though high-performing students usually are less willing to initiate friendship and advice relationships with low-performing students [25], they may be more willing to do this in the mixed international LCs, since they want to fit in, grasp the local language and adapt to the local culture and local medical system. Therefore, the high-performing students in mixed international LCs may keep more study-support relationships and friendships with other students than those in domestic LCs. Thus, the difference of students’ interpersonal relationships might also contribute to the finding that students in mixed international LCs outperformed the students in domestic LCs in the end.

### Strengths and limitations of the study, further research and implications for practice

One strength of this study is that this is the first study, as far as we know, that studied the influence of mixing international students with domestic students in small group learning on their competency learning. Furthermore, the fact that we were not only able to compare students’ competencies performances over the first two years of their medical study but also that the findings over a long period of time (two years) stayed consistent, strengthens our results. Additionally, all students in the different LCs followed the same basic programme, so differences in curriculum design cannot explain the observed differences.

We considered two important limitations of our study. First, we only analyzed the assessment of three competencies (i.e., Collaboration, Leadership, Professionalism) rather than all seven competencies in the CanMEDS framework (i.e., Medical expertise, Communication, Collaboration, Leadership, Health advocacy, Scholarship, and Professionalism [28–34]). These competencies were selected because they were assessed within the shared programme and identical for all students in all LCs. In order to minimize confounding differences in educational programme and teacher staff, we have only included similar parts of the programme. Therefore, further studies should explore to what extent the other four competencies have their special characteristics and can be assessed within the different LC programmes.

Second, our study makes use of the three-scale scoring system for the assessment of competencies. These kinds of longitudinal programmatic assessments in a large number of students give a detailed insight how competencies develop over time. This three-scale scoring reduces the variance when analyzing differences in assessment for students in the different groups. The Spearman rank correlation can result in a bias. However, Zimmerman et al. (2003) showed with simulation studies that the bias might be limited when the sample size is large enough [49].

Future research should include more qualitative studies to investigate why students in the mixed, international and domestic groups outperformed in this study. Based on that, we can come up with better ideas on how to improve medical students’ competencies learning. For instance, it is important to know if students in such combined groups have higher academic motivation since motivation is a critical influence factor of students’ academic performance [50]. Studies should include exploring students’ intrinsic and controlled motivation in mixed international group learning, motivation differences among groups and its development within groups over time, and the relationship between students’ motivations and attitudes to the assessment system and the way of grading.

Moreover, it is worthwhile to know how students’ social networks develop and compare that among different group compositions [47]. Next to the formal learning network as organized by the faculty, the informal learning network may also crucially influence students’ academic achievement [51]. This may help us to know if and to what extent students’ interpersonal relationships and informal learning influence their competency learning, and how to improve formal curricula design.

## Conclusion

This explorative study indicates that when international students are introduced in small groups with domestic students, all students meet the basic level of the key competencies (Collaboration, Leadership, Professionalism). Importantly, students in the mixed international and domestic LCs outperformed students in homogenous domestic groups. The differences were not caused by the background of the students. Differences in motivation and differences in social networks of the students among mixed international LCs might explain this finding. Further research is needed to explore this phenomenon.

## Data Availability

The dataset used and analyzed during this study is available from the corresponding author upon reasonable request.

## Appendix

**Table 3:** Spearman rank correlation of Fast-on-track in three competencies and background characteristics

	Collaboration	Leadership	Professionalism	First progress test	Age	Gender	International students
Collaboration	1						
Leadership	.493^a^	1					
Professionalism	.496^a^	.528^a^	1				
First progress test	.054	.064	.073	1			
Age	.067	.054	.039	−.020	1		
Gender	−.013	−.006	.009	.048	−.176^a^	1	
International students	.068	.104^a^	.158^a^	−.016	.308^a^	−.138^a^	1

^a^The Spearman rank correlation is significant at the 0.01 level (2-tailed)

## Abbreviations

PBL: Problem-based learning
CBME: Competency-based medical education
LC: Learning community
